# Improving Asthma during Pregnancy with Dietary Antioxidants: The Current Evidence

**DOI:** 10.3390/nu5083212

**Published:** 2013-08-14

**Authors:** Jessica A. Grieger, Lisa G. Wood, Vicki L. Clifton

**Affiliations:** 1 Robinson Institute, School of Paediatrics and Reproductive Health, Adelaide University, Lyell McEwin Hospital, Haydown Road, Elizabeth Vale, SA 5112, Australia; E-Mail: jessica.grieger@adelaide.edu.au; 2 Hunter Medical Research Institute, School of Biomedical Science and Pharmacy, University of Newcastle, Kookaburra Circuit, New Lambton Heights, NSW 2305, Australia; E-Mail: lisa.wood@newcastle.edu.au

**Keywords:** asthma, pregnancy, nutrition, antioxidants, oxidative stress

## Abstract

The complication of asthma during pregnancy is associated with a number of poor outcomes for the mother and fetus. This may be partially driven by increased oxidative stress induced by the combination of asthma and pregnancy. Asthma is a chronic inflammatory disease of the airways associated with systemic inflammation and oxidative stress, which contributes to worsening asthma symptoms. Pregnancy alone also intensifies oxidative stress through the systemic generation of excess reactive oxidative species (ROS). Antioxidants combat the damaging effects of ROS; yet antioxidant defenses are reduced in asthma. Diet and nutrition have been postulated as potential factors to combat the damaging effects of asthma. In particular, dietary antioxidants may play a role in alleviating the heightened oxidative stress in asthma. Although there are some observational and interventional studies that have shown protective effects of antioxidants in asthma, assessment of antioxidants in pregnancy are limited and there are no antioxidant intervention studies in asthmatic pregnancies on asthma outcomes. The aims of this paper are to (i) review the relationships between oxidative stress and dietary antioxidants in adults with asthma and asthma during pregnancy, and (ii) provide the rationale for which dietary management strategies, specifically increased dietary antioxidants, might positively impact maternal asthma outcomes. Improving asthma control through a holistic antioxidant dietary approach might be valuable in reducing asthma exacerbations and improving asthma management during pregnancy, subsequently impacting perinatal health.

## 1. Introduction

### 1.1. Asthma

Allergic diseases such as asthma, hay fever, and rhinitis are amongst the most common chronic diseases affecting people in developed countries [1,2,3,4]. Over the last 40 years, there has been an increase in the prevalence of allergic diseases, particularly in Western countries, with about 300 million people suffering from asthma [1,5]. The financial cost of asthma is high. In 2004–2005, $606 million was spent on asthma in Australia, with more than half spent on medication [6].

Asthma prevalence in Australia is one of the highest in the world, with a prevalence of ~10% in adults [7]. By the age of four to five years, nearly a quarter of children have reported asthma or wheeze at some stage, with diagnosis of asthma in 11.5% [6]. The likelihood of having asthma in childhood is increased when the mother has asthma [8], suggesting the disease has an intergenerational impact that may not be related to genetic susceptibility alone.

### 1.2. Asthma in Pregnancy

Asthma is the most prevalent chronic medical disease to complicate pregnancies worldwide, with a prevalence in pregnancy of 8%–13% [9,10,11]. In Australia, it affects around 12% of pregnant women or 36,000 pregnancies each year [12]. Asthma in pregnancy has been shown to be associated with a number of poor outcomes for the mother and fetus. In a retrospective cohort study from 223,512 singleton deliveries from 12 clinical centres across the United States, it was found that pregnant women with asthma were more likely to develop preeclampsia (adjusted odds ratio (aOR) 1.14; 95% CI: 1.06–1.22), gestational diabetes (aOR 1.11; 95% CI: 1.03–1.19), and delivering preterm (aOR 1.17; 95% CI; 1.12–1.23) [13]. In a retrospective study from Australia (*n* = 16,672), pregnant women with asthma had smaller babies (3294 g *vs*. 3353 g), a higher percentage were smokers (34% *vs*. 28%), and a higher percentage had essential hypertension (3% *vs*. 2%) compared to non-asthmatics [14]. In addition, male neonates of asthmatic mothers were more likely to be small for gestational age (SGA) and have smaller head circumference than those of the non-asthmatic mothers [14]. In a meta-analysis of adverse perinatal outcomes among women with asthma, the presence of asthma in pregnancy was associated with an increased relative risk (RR) of low birth-weight (RR 1.46; 95% CI 1.22–1.75), SGA (RR 1.22; 95% CI: 1.14–1.31), preterm delivery (RR 1.41; 95% CI: 1.22–1.61), and pre-eclampsia (RR 1.54; 95% CI: 1.32–1.81) [15]. Maternal asthma has also been associated with bronchiolitis in infancy [16], childhood asthma [17,18], and atopy in children [19].

Asthma exacerbations are common in pregnancies complicated by asthma affecting at least 35% to 55% of pregnancies [20,21]. This particular event has been shown to be associated with a greater risk for low birth-weight (RR 3.02; 95% CI: 1.87–4.89) and preterm delivery (RR 1.54; 95% CI: 0.89–2.69) [22] suggesting the management of asthma during pregnancy is essential to avoid asthma exacerbations and for improved fetal outcomes.

## 2. Pathophysiology of Asthma

Asthma is a complex inflammatory disease of the airways with symptoms including excess mucus production, wheeze, dyspnoea, cough, fatigue, anxiety, tachycardia, and chest tightness [23,24]. The acute inflammatory response induces asthmatic symptoms such as mucus hypersecretion, plasma exudation, and bronchoconstriction [23,25], and may lead to reversible airway obstruction. However, as the disease progresses, persistent airway inflammation leads to repeated attacks, bronchial constriction and hyper-responsiveness [26]. This is characterised by long-term airway remodeling, with sub-epithelial fibrosis, epithelial cell injury, mucus hypersecretion, angiogenesis, and smooth muscle hypertrophy and hyperplasia [23,25].

A central feature of asthma is inflammatory cell infiltration of the respiratory tract by neutrophils, eosinophils, mast cells [27], and lymphocytes [23]. Airway inflammation in asthma is heterogeneous and may be dominated by an allergen-specific acquired immune response with IL-5 mediated eosinophilic inflammation, or a dysregulation of innate immune responses involving IL-8-induced neutrophilic airway inflammation [28,29]. Importantly, both pathways lead to the production of reactive oxygen species (ROS). The continuous exposure of the respiratory tract to environmental oxidants and airway inflammatory cell-generated ROS creates a high level of oxidative stress in the lung [30,31].

### 2.1. Oxidative Stress

Activation of inflammatory cells such as mast cells, macrophages, eosinophils, netrophils, lymphocytes, and platelets in the airways [25,32] by stimuli such as allergens, viruses, endotoxin, and smoking, leads to a respiratory burst [33,34] of activated inflammatory cells with increased oxygen consumption [35]. Experimental studies in humans have demonstrated that this respiratory burst can generate various ROS such as superoxide anions, hydrogen peroxide, and hydroxyl radicals, leading to increased quantities of free radicals both in the blood and airway tissues [36,37,38,39]. Cell culture studies in humans and animals have shown ROS can amplify airway inflammation by activating redox-sensitive transcription factors such as NF-κB [40] and JAK-STAT [41], and hydrogen peroxide can activate extracellular signal-regulated kinase Raf-1 [42], leading to further amplification of the transcription of pro-inflammatory genes. This increase in inflammation subsequently impacts the airways [43]. Evidence of oxidative stress in the airways in asthma has been demonstrated by increased exhaled nitric oxide [44,45,46], increased exhaled lipid peroxidation products such as thiobarbituric acid-reactive substances [47], and increased superoxide anion radicals in bronchoalveloar lavage fluid in asthmatics compared to controls [48]. The redox imbalance is not only confined to the lungs but measurable systemically in asthma (e.g., plasma isoprostanes and malondialdehyde) [49,50,51]. Oxidative stress markers have also been shown to be increased during acute asthma exacerbation in adults [52,53], during exacerbation in children [54], and highest in those with severe asthma [55]. Managing asthma and asthma control through minimising oxidative damage appears a necessary factor for reducing asthma severity.

### 2.2. Pregnancy, Oxidative Stress, and Asthma

Pregnancy is a stressful condition in which many maternal physiological and metabolic functions are altered. In normal human pregnancies, there is increased production of superoxide anions in early gestation [56,57,58,59] and which continues throughout gestation [60,61]. This increased oxidative stress is considered a normal part of pregnancy and levels return to normal post-partum [61]. At this time, an increase in a variety of antioxidant mechanisms controls redox balance [62,63]. However, when the oxidant-antioxidant balance is offset by either heightened oxidative stress or limited antioxidant capacity, excess ROS can contribute to clinical symptoms of gestational diabetes [64], pre-eclampsia [65,66], and asthma [67]. In addition to systemic oxidative stress, oxidative stress manifests at the maternal-fetal interface and contributes to normal placental development. Maternal circulation to the placenta is only fully established at 10–12 weeks of gestation [68], and as a result, leads to high rates of ROS [69,70]. Placental antioxidant enzymes and associated factors are increased [62,71,72], possibly serving as protection of the fetus and the maintenance of pregnancy.

The adverse effects of asthma on the fetus may be due, in-part, to the increase in oxidative stress. Experimental evidence for heightened oxidative stress in pregnancies complicated by asthma was first shown by Clifton *et al*., in which placentae of pregnant women with asthma had higher levels of lipid peroxidation markers and oxidative stress markers compared to placentae of women without asthma [67]. Later, increased placental cytokine mRNA expression was also identified in placentae of pregnancies complicated by mild- and moderate-severe asthma, compared to placentae from non-asthmatic women [73].

Oxidative stress increases in the presence of asthma exacerbations [53] but it has not been examined in exacerbations during pregnancy. The effects of oxidative stress produced by the complication of asthma and asthma exacerbation during pregnancy could potentially be alleviated through an increase in antioxidant supplementation.

## 3. The Role of Antioxidants in Asthma

The lungs have endogenous antioxidant mechanisms to combat the damaging effects of ROS. The major enzymatic antioxidants in the lungs are superoxide dismutase, glutathione peroxidase and catalase [74]. Important non-enzymatic antioxidants include vitamin E, vitamin C, albumin, uric acid, ceruloplasmin, and glutathione [75,76,77]. Some studies have demonstrated that levels of antioxidants in the lungs and circulation are reduced in asthmatics. In asthmatic adults, induced sputum carotenoid levels were lower compared to carotenoids measured in whole blood and plasma, and total- and individual carotenoids were lower in whole blood in asthmatic adults compared to healthy controls [78]. Further, Wood *et al*. identified that adults with airway hyperresponsiveness had reduced levels of whole blood β-carotene, α-tocopherol, and total tocopherols compared with those without airway hyperresponsiveness [79]. Those with severe-persistent asthma had reduced whole-blood levels of α-tocopherol compared with those with mild-moderate asthma; and in those with stable, but poorly controlled asthma, plasma antioxidant potential was lower compared with those with controlled or partly controlled asthma [79]. Other studies have also found that antioxidant concentrations both systemically and in the lungs are consistently reduced in asthmatic patients [48,80,81,82,83], as well as during asthma exacerbations in both adults [52] and children [54]. Oxidative stress is evident in asthma and antioxidant status, measured both systemically and in the lungs, is compromised. Hence, balancing the oxidant-antioxidant system may prove to be an appropriate target for ameliorating oxidative stress in asthma.

Only two separate studies in human asthma research have reported on antioxidant status in pregnancy. First, the study by Clifton *et al*. reported that antioxidant enzyme activity in the placenta was up-regulated in pregnancies complicated by asthma compared to non-asthmatics [67]. A later study identified that pregnant women with moderate-severe asthma had increased plasma concentrations of total carotenoids and other dietary antioxidants late in gestation, compared to those women with mild asthma and healthy pregnant controls [84]. Moreover, pregnant asthmatic women with low circulating concentrations of various antioxidants had poorer fetal growth outcomes such as head circumference and birth weight. This suggests there is a compensatory increase in antioxidant production and distribution to protect the fetus from the increased oxidative stress that occurs with the combination of asthma and pregnancy.

### 3.1. The Potential for Dietary Antioxidants to Improve Asthma

Dietary/non-enzymatic antioxidants are the first line of defence against ROS and include vitamin C, lycopene, γ-tocopherol, α-tocopherol, selenium, carotenoids and flavonoids. Table 1 describes some food sources of various antioxidants found in the diet, as well as their function relevant to asthma. In short, α-tocopherol acts to break free radical chain reactions involved in lipid peroxidation [85], thus converting the peroxyl and oxidative radicals to less reactive forms [86], and maintaining fatty acid membrane integrity [87]. As such, increased α-tocopherol might provide protection against oxidative damage to the lungs [86]. α-tocopherol has been shown to inhibit markers of inflammation in human lung epithelial A549 cells, thereby potentially acting as an anti-asthma agent [88]. Carotenoids are fat soluble phytochemical plant pigments which include α- and β-carotene, lutein, lycopene, and β-cryptoxanthin [89]. Twenty four carotenoids have been reported to provide health benefits due to their antioxidant properties [90]. In particular, lycopene and β-carotene have been shown to scavenge ROS [91,92]. An increase in dietary antioxidants may improve asthma during pregnancy, however, further studies are required.

**Table 1:** Dietary antioxidants, food sources, and their function relevant to asthma.

Antioxidant	Fruits	Vegetables	Other food sources	Function relevant to asthma
Vitamin C	Orange, kiwi fruit, grapefruit, apricot.	Potato, red capsicum, snowpeas, broccoli, spinach.		Accelerates histamine metabolism, direct effects on smooth muscle, and cyclic adenosine monophosphate [93]. Vitamin C has been shown to block TNF-α mediated activation of the transcriptional factor NF-κB, a key regulator of inflammation [94].
Vitamin E			Almonds, vegetable oils, meat, poultry, nuts, eggs.	Increases COX-2 activity and prostaglandin E-2 production by macrophages, promoting the differentiation of T cells to Th-2 lymphocytes [95]; directly affects T cells by down-regulating the expression of interleukin-4 mRNA in T-cells [96]. Suppresses neutrophil migration and inhibits IgE production [75,97].
Carotenoids (*i.e.*, β-carotene, α-carotene, γ-carotene, lycopene, β-cryptoxanthin, and lutein-zeaxanthin)	Orange, pumpkin, sweet potato.	Carrots, sweet potatoes, spinach, tomato, pumpkin, red capsicum, juice, tomato juice, carrot juice.	Pistachio nuts.	Antioxidant activities, participation in cell signaling pathways, and decreasing inflammation [98].
Flavonoids (*i.e.*, catechins, quercetin, epicatechin)	Citrus fruits.	Tomato, red onion, onion.	Tea (green), cocoa, red wine.	Scavenge nitric oxide [99]; inhibit release of histamine, arachidonic acid, and production of cytokines [100].

Antioxidant food sources identified from NUTTAB 2010 online database (except flavanoids [101]).

### 3.2. Epidemiologic Studies Assessing Antioxidant-Rich Foods and Antioxidant Intakes and Asthma

Epidemiologic studies suggest that the daily intake of antioxidant-rich fruits and vegetables in Australia are below recommended levels. In the most recent Australian Health Survey it was reported that 48.3% of Australians aged 18 years and over “usually” met the guideline for daily fruit intake (two servings or 300 g/day), while only 8.3% met the guideline for daily vegetable intake (five servings or 375 g/day) [102]. Overall, 3.0% of Australians aged 25–34 years consumed the recommended intake of fruit and vegetables, compared to 9.6% of those 65–74 years [102]. Similar results were found globally: Currently in the USA, just under 50% of the population consume ≥2 servings/day of fruit or ≥3 servings/day vegetables [103], while in Europe, the mean vegetable intake (including pulses and nuts) is 220 g/day and the mean fruit intake is 166 g/day, with >50% of the countries of the World Health Organisation European Region consuming lower than 400 g/day of fruit and vegetables [104]. Nationwide, it is evident that consumption of antioxidant-rich fruits and vegetables is poor and further strategies aimed at increasing intakes are warranted. Dietary intakes of specific antioxidant vitamins such as vitamin C generally meet recommendations [105], however tend to be lower for vitamin A and for vitamin E [106,107,108].

A number of epidemiologic studies have assessed dietary antioxidant intakes and their association with asthma, asthma control, wheeze, bronchitis and atopy in adults. However evidence from clinical trials demonstrating an improvement in asthma is lacking. Data from adults participating in the first National Health and Nutrition Examination Survey (NHANES 1) (1971–1974) identified that there were significant differences in lung function (*i.e.*, lower forced expiratory volume in one second (FEV_1_)) (2530 mL *vs*. 2550 mL *vs*. 2570 mL) in those with lower vitamin C intake (≤17 mg *vs*. 66 mg/day *vs*. ≥178 mg/day) [109]. Other studies have reported mixed results between vitamin C intake and lung function (as measured by FEV_1_ or forced vital capacity (FVC)) [110,111,112,113,114,115]. In multivariate analyses, a number of studies have reported no significant associations between intakes of vitamin E [111,113,114,115,116] or β-carotene [110,111,115,116] and FEV_1_. Table 2 describes the studies in adults in which higher intakes of the antioxidant vitamins C, E and A, were positively associated with increased FEV_1_. In NHANES III, assessment of all nutrient intakes revealed that for every one standard deviation increase in vitamin C (113 mg/day), vitamin E (9.1 mg/day), and β-carotene (1170 retinol equivalents/day), there was an increase in FEV_1_ of 24.6 mL [117], which was higher than all nutrients assessed individually. This suggests that it is the synergistic effects of all the antioxidants that improve lung function and supplementation with an individual antioxidant may not be of benefit. One study assessing dietary carotenoid intakes reported no association between cryptoxanthin or lycopene and FVC or FEV_1_, however vitamin E was correlated with FEV_1_, and lutein-zeaxanthin was correlated with FVC% [118]. There is inconclusive evidence for an association between vitamins C, E, and A intakes and atopy [97,116] or wheeze [116,119] but vitamin C may be beneficial in bronchitis [119]. In a population-based cohort from the UK, dietary vitamin C and E intakes were not associated with asthma [114]; nor was there a positive effect of dietary antioxidant intake and lung function/asthma in a meta-analysis of seven observational studies [120]. Furthermore, one study in Dutch adults reported negative findings, such that dietary intakes of vitamin E and β-carotene were associated with cough (OR: 1.26; 1.02–1.56) and wheeze (OR: 1.27; 1.04–1.55), respectively [113].

Most of the observational evidence points towards a positive effect of higher vitamin C intake and lung function but not for reducing risk of asthma. It appears that for an approximate 88 mg/day increase in vitamin C, there is an associated increase in FEV_1_ of 31 mL, and for vitamin E, an increase in 3.6 mg/day appears to be associated with a 28 mL increase in FEV_1_. To observe this effect on FEV_1_ with vitamin C and vitamin E intake, respectively, this would be equivalent to eating four apricots/day or 22 almonds (25 g/day). A change of >12% or 200 mL FEV_1_ asthmatic patients has been suggested a clinically significant change in lung function [121,122]. Dietary intakes of antioxidant vitamins would therefore need to be increased further to produce a clinically significant change.

**Table 2:** Cross-sectional associations between dietary intakes of antioxidant vitamins C, E, and A, and lung function in adults with asthma.

Author, country, population	Age range	*n*	Mean intake/day	Change in intake	Effect of change in intake on FEV_1_ (predicted difference: 95% CI)	Effect of change in intake on FVC (predicted difference: 95% CI)
**Vitamin C**						
McKeever *et al.*, UK, general population [116]	27–80 years	1346	Not reported	↑ 100 mg/day	66.8 (12.2–121.4) mL	Not measured
Hu *et al*., 69 countries in rural China [123]	35–64 years	3085	151 mg	↑ 100 mg/day	21.6 (−0.4–43.5) mL	24.9 (0.2–49.6) mL
Hu *et al*., USA, NHANES general population [117]	≥17 years	16,693	111 mg	↑ 1 SD (113 mg/day)	9.5 (−0.2–19.2) mL	Not measured
Britton *et al*., UK, general population [124]	18–70 years	2633	99.2 mg	↑ 1 SD (40 mg/day)	25 (5.2–44.8) mL	23.3 (0.94–45.7) mL
**Vitamin E**						
Butland *et al*., UK, men [110]	45–59 years	2512	51.4 mg	↑ 1 SD (2 mg/day)	31.7 (0.9–62.5) mL	Not measured
Hu *et al*., USA, NHANES general population [117]	≥17 years	16,693	9.2 mg	↑ 1 SD (9.1 mg/day)	16.4 (5.5–27.4) mL	Not measured
Britton *et al*., UK, general population [124]	18–70 years	2633	6.2 mg	↑ 1 SD (2.2 mg/day)	20.1 (1.3–40.4) mL	23.1 (1.0–45) mL
Dow *et al*., UK, general population [112]	70–96 years	178	5.3 mg (median)	↑ 1 mg/day	42 (39–45) mL	53 (18–88) mL
**Vitamin A**						
Hu *et al*., USA, NHANES general population [117]	≥17 years	16,693	567 µg	↑ 1 SD (1107 µg/day)	18.2 (8.7–27.6) mL	Not measured

↑: increase.

There have been few studies assessing antioxidant-rich fruit and vegetable intake on asthma and lung function. From the three studies identified, there is inconclusive evidence indicating higher fruit intake over five years [110] or higher fruit and vegetable intake over 10 years [115] is associated with increased FEV_1_. However, a large decrease in fruit consumption over seven years was associated with decreases in FEV_1_ by approximately 89 mL/year and 133 mL/year, respectively, compared with those with stable fruit consumption [125]. Consumption of ≥2 apples/day was associated with an increase in FEV_1_ of 138 mL compared to no consumption of apples [110]. Fruits and vegetables contain a range of antioxidant vitamins and carotenoids, and although a couple of studies indicate an improvement in asthma control with higher consumption, further studies assessing fruit and vegetable intake in asthmatic adults are needed. These studies would be of particular importance as, in addition to antioxidant vitamins, fruits and vegetables are high in fibre and low in fat. Importantly, lower fibre, higher fat diets have also been linked to worse airway inflammation and lung function in severe asthmatics [126]. Altering food intake patterns towards an antioxidant diet, which is also fibre-rich and low in fat, might be protective in asthma.

### 3.3. Randomized Controlled Trials Assessing Antioxidant Supplements in Adults with Asthma

Given the number of epidemiologic studies assessing the effect of antioxidant intakes on asthma and lung function, there have been relatively few supplementation studies (Table 3). The few available studies provide inconsistent evidence to support antioxidant use to improve asthma. At present, there is little evidence to recommend a specific role for vitamin C in the treatment or management of asthma. Importantly, the majority of the studies that were included in a Cochrane review [127] were of poor quality and several studies were not included in the analysis due to no placebo group, non-RCT design, or because the vitamin C was combined with other/non-antioxidant micronutrients.

For β-carotene, there is some evidence demonstrating a protective role against exercise induced asthma [128]. For vitamin E, there was no effect of 500 mg/day vitamin E for six weeks on any measure of asthma control [129]. One short-term (seven day) study assessing lycopene supplementation, reported no clinical effects on asthma following 45 mg/day lycopene, however, increases in plasma carotenoid concentrations and decreased percentage of neutrophils in sputum were observed [130]. There is mixed evidence for one week of lycopene supplementation (30 mg/day) on lung function after exercise [131,132]. Only one RCT has examined the effect of manipulated antioxidant intake through changing intake of whole foods on asthma outcomes and lung function in asthmatic adults [133]. Specifically, compared to a high fruit (≥2 servings/day) and vegetable (≥5 servings/day) diet for 14 weeks, the asthmatic subjects who consumed a low fruit (<1 serving/day) and vegetable (<2 servings/day) diet had a 2.26 increased risk of asthma exacerbation [133].

To date, supplementation with individual antioxidants has not been shown to be beneficial in improving lung function or asthma control in adults with asthma. This is supported by two recent reviews indicating that there appears to be little clinical support for dietary antioxidant supplementation in asthma [134,135]. Although some positive studies were found using supplementation with β-carotene and lycopene, further studies are required to support this. Supplementation with vitamin E does not appear beneficial. Changing the dietary intake to antioxidant-rich fruit and vegetables, rather than supplementation, was an effective approach for reducing asthma exacerbations. Further studies to confirm the use of dietary manipulation as a management strategy for asthma are warranted.

**Table 3:** Intervention studies assessing antioxidant supplementation on asthma outcomes in adults with asthma.

Study population	Design	Intervention	Outcomes
Vitamin C			
Cochrane Review in adults and/or children with chronic stable asthma, seasonal asthma, or EIA [127]	Review: 9 RCTs (*n* = 330). 3 parallel studies, 6 cross-over studies. Only 5 studies contributed numerical data.	3 studies used long-term supplementation: 1 g/day vitamin C for 14 weeks, 6 months and 16 weeks; 500 mg/day vitamin C for 7 days; 1500 mg over 2 weeks. 4 studies used single doses of vitamin C (2 g, 2 g, 2 g and 500 mg).	**Primary outcomes** **FEV_1_%:** - Change in FEV_1_ (L)—post-exercise challenge: (MD 0.13; 95% CI −0.05–0.31) [136]. - Significant ↓ intervention post-exercise (−6.4 ± 2.4%) *vs*. usual (−14.3 ± 1.6%) and placebo (−2.9 ± 2.4%) [137]. - FEV_1_ (mL) at 4 months: (MD −11.00; 95% CI −91.36–69.36) [138]. **FVC:** - Change in FVC (L)—post-exercise challenge: (MD 0.13; 95% CI −0.03–0.29) [136]. **Secondary outcomes:** - Significant improvement in asthma symptom scores (Asthma Quality of Life Questionnaire): Intervention (6.3; 95% CI 5.8–6.8) *vs*. placebo (5.8; 5.1–6.2) *vs*. usual (5.6; 5.0–6.3) [137]. - IgE (IU/mL serum): absolute values at 1 month: (MD 4.00; 95% CI −140.42–148.42) 3 months (MD −312.00; −628.21–4.21) and 6 months (MD −143.00; −425.38–139.38) [139]. - NS for inhaled corticosteroid use [138].
β-carotene			
38 patients with EIA [128]	Randomized, double-blind, placebo controlled trial.	64 mg/day β-carotene *vs*. placebo for 1 week.	- All 38 patients taking placebo revealed a significant post-exercise reduction of more than 14% in their FEV_1_. - 20 (53%) of the 38 patients with daily β-carotene supplementation were protected against EIA.
Vitamin E			
72 participants from a clinical trial register of 18–60 year olds with asthma [129]	Randomized, placebo controlled, double blind parallel group clinical trial.	500 mg/day natural vitamin E or matched placebo for 6 weeks.	- No effect of vitamin E supplementation on measures of asthma control (FEV_1_, FVC), mean morning and evening peak flow, symptom scores, bronchodilator use, or serum IgE.
Lycopene			
32 asthmatic adults, 52 years of age [130]		Low antioxidant diet for 10 days, then commenced a randomized, cross-over trial involving 3 × 7 day treatment arms: placebo, tomato extract (45 mg/day lycopene) and tomato juice (45 mg/day lycopene).	Low antioxidant diet: Plasma: - 42% ↓ in plasma lycopene. - NS for α- and γ-tocopherol. Asthma control - ↓ % predicted FEV_1_: 79.4 (71.6–87.2) *vs*. 76.5 (68.9–84.1). - ↓ % predicted FVC: 93.0 (87.1–98.9) *vs*. 90.4 (84.3–96.5). - ↓ Asthma Control Score: 1.0 (0.6–1.4) *vs*. 1.4 (1.0–1.8). - ↑ % neutrophils in induced sputum: 31.0 (13.1–45.9) *vs*. 41.0 (24.2–56.6). Supplementation: - Significant ↑ in plasma carotenoid concentrations. - ↓ in % neutrophils (55% *vs*. 42%). - NS for % predicted FEV_1_, % predicted FVC or FeNO. - NS for asthma control score.
19 adults with exercise-related difficulty in breathing [131]	Randomized cross-over study, with 2 weeks wash-out.	30 mg/day lycopene *vs*. placebo for 1 week.	Asthma control - NS in FEV_1_ after exercise with lycopene treatment *vs*. placebo (12% *vs*. 11%).
20 patients with EIA [132]	Not reported if randomized. Double blinded.	30 mg/day lycopene *vs*. placebo for 1 week.	- Lycopene for 1 week significantly protected against EIA in 11 of 20 patients. - All 20 patients taking placebo revealed a significant post-exercise reduction of more than 14% in their FEV_1_.

FEV_1_: forced expiratory volume in 1 s; MD: mean difference; IgE: Immunoglobulin E; NS: not significant; EIA: exercise induced asthma; FVC: forced vital capacity; ↑: increase; ↓: decrease; FeNO: fractional exhaled nitric oxide.

### 3.4. Antioxidants in Pregnancies Complicated by Asthma

Dietary energy and nutrient requirements are generally increased during pregnancy [105,140]. As such, maternal nutrition has the potential to influence fetal growth and development. Over the past 20 years, there has been a shift from higher consumption of traditional plant based foods to diets higher in processed foods [5,102,141,142]. This increased intake of processed foods and higher intake of fat, specifically saturated fat, and sugar, parallels the reduced intake of fruits and vegetables that contain antioxidants [143,144,145]. Current evidence indicates that in normal pregnancy, despite sufficient maternal energy and macronutrient intakes, several key maternal micronutrients including folate, iron, zinc, calcium, vitamin D and essential fatty acids are less than optimal [84,146,147,148]. Regarding dietary antioxidant intakes, in a sample of 300 pregnant women from Perth, Western Australia, with family history of allergic disease, dietary intakes of vitamin C, β-carotene, selenium and copper were generally greater than the recommended dietary intake, however intakes of vitamin E and zinc was lower [149]. Dietary intake of vitamin C during the first and second trimesters was higher than US recommendations, with vitamin E intake at the recommended intake [95]. Similar intakes of vitamin C (174 mg) and vitamin E (8.4 mg) were reported in 1704 pregnant mothers in the UK [150].

There has been only one study assessing antioxidant intakes in pregnant women with asthma in which there was no significant difference in antioxidant intakes between women with moderate-severe asthma (*n* = 34), mild asthma (*n* = 25) or controls (*n* = 32) [84]. However, in that study, in the asthmatic women, low systemic antioxidant concentrations were associated with reduced fetal growth, whereas in non-asthmatics, no such association was found [84], suggesting the need to improve antioxidant status in pregnant asthmatic women. In an eight-week pilot study in pregnant women with history of asthma, a food-based exchange intervention to increase vitamin E containing foods was reported to be feasible and acceptable [151]. However, asthma outcomes and antioxidant concentrations were not reported in that publication. There is currently no published data on the relationship between asthma in pregnancy and how dietary antioxidant intakes modify circulating antioxidant concentrations. The introduction of an antioxidant-rich diet to pregnant asthmatic women may be of benefit to maternal asthma severity and fetal growth and development; studies in this area need to be conducted.

## 4. Current Evidence

Although there has been a number of reviews on diet and asthma in the past five years, these reviews focused on the impact of foods and nutrients on the risk of children developing allergies such as wheeze and asthma [1,152] either perinatally [153,154], or postnatally [154], or reviewed the evidence on anti-inflammatory oils [155,156]. Collectively, two further reviews reporting on nutrition and asthma [135] and on antioxidants and allergic disease [134] concluded that despite some relationships observed between antioxidants and asthma, further high quality research on asthma in adults is required to disentangle the effects of nutrition, specifically antioxidants, on asthma outcomes. This current review echoes these findings and updates the previous literature with additional recent publications. A recently published randomized controlled trial by Wood *et al*. [133] demonstrated the positive effects of a high fruit and vegetable diet on asthma control; however additional intervention studies are required to support this.

Regarding antioxidants and asthma in pregnancy, the work by Scott *et al*. [73], McLernon *et al*. [84], and Murphy *et al*. [15] provides the most up-to-date work on this topic and provides compelling information regarding altered oxidant-antioxidant mechanisms in pregnant women with asthma, as well as the impact of poor asthma control in pregnancy on perinatal outcomes. The information reported in this review suggests the need for dietary interventions to support the prospective relationships observed in those studies.

## 5. Conclusions

Both asthma and pregnancy are associated with increased oxidative stress. The increased oxidative load with asthma suggests that manipulation of dietary antioxidants might be important. Currently however, there are few randomized controlled trials in asthmatic subjects to confirm this, and only one study revealed that a diet high in antioxidants improved asthma outcomes in adults. Importantly, this was achieved through a whole food approach rather than a single antioxidant supplement. It is established that asthma in pregnancy affects maternal and neonatal health; oxidative stress may be a key player promoting these adverse outcomes. Pregnancy is considered a key time for dietary modification to support fetal development. Nutritional strategies that can lower maternal oxidative stress during pregnancy may also improve pregnancies complicated by asthma.
